# Supplementary material for: Telomere length is associated with growth in children in rural Bangladesh
Source: eLife. 2021 Sep 8;10:e60389. doi: 10.7554/eLife.60389 (PMC8494482; doi:10.7554/eLife.60389)
Supplement: Supplementary file 1. — (a) Pre-specified covariates screened for inclusion in fully adjusted models. (b) Association Between Telomere Length at Year 1 and Growth. (c) Association Between Telomere Length at Year 2 and Growth. (d) Association Between Change in Telomere Length and Growth. (e) Post-hoc Analyses: Association Between Growth at Month 3 and Subsequent Telomere Length. (f) Post-hoc Analyses: Association Between Growth at Year 1 and Subsequent Telomere Length. (g) Post-hoc Analyses: Association Between Change in Growth and Telomere Length. (h) Post-hoc Analyses: Association Between Growth Velocity and Telomere Length. [file elife-60389-supp1.docx]

**Supplementary file 1 – Supplementary Tables**

**Table of Contents**

Supplementary file 1a: Pre-specified covariates screened for inclusion in fully adjusted models 2

Supplementary file 1b: Association Between Telomere Length at Year 1 and Growth 3

Supplementary file 1c: Association Between Telomere Length at Year 2 and Growth 5

Supplementary file 1d: Association Between Change in Telomere Length and Growth 6

Supplementary file 1e: Post-hoc Analyses: Association Between Growth at Month 3 and

Subsequent Telomere Length 7

Supplementary file 1f: Post-hoc Analyses: Association Between Growth at Year 1 and

Subsequent Telomere Length 8

Supplementary file 1g: Post-hoc Analyses: Association Between Change in Growth and

Telomere Length 9

Supplementary file 1h: Post-hoc Analyses: Association Between Growth Velocity and

Telomere Length 11

References 12

**Supplementary file 1a. Pre-specified Covariates Screened for Inclusion in Fully Adjusted Models**

^a^The caregiver-reported diarrhoea covariate was measured at Years 1 and 2 and defined as at least 3 loose or water stools within a 24-hour period or at least 1 stool with blood.^1^

^b^The maternal depression covariate was assessed using the Center for Epidemiologic Studies Depression Scale (CESD-R) at Years 1 and 2.^2^ The cut point for clinical depression on the CESD-R scale is 16.

^c^The maternal stress covariate was assessed using the Perceived Stress Scale (PSS) measured at Year 2.^3, 4^

^d^The maternal lifetime exposure to physical, sexual, or emotional intimate partner violence covariate was measured using the WHO Health and Life Experiences Survey administered at Year 2.^5^

^e^Assessed by the Household Food Insecurity Access Scale (HFIAS).^6^

**Supplementary file 1b. Association Between Telomere Length at Year 1 and Growth**

| Exposure | Outcome | N | 10th Percentile | 90th Percentile | Outcome, 90th Percentile v. 10th Percentile | | | | | | | | | | |
| --- | --- | --- | --- | --- | --- | --- | --- | --- | --- | --- | --- | --- | --- | --- | --- |
|  |  |  |  |  | Unadjusted | | | | | Adjusted^a^ | | | | | |
|  |  |  |  |  | Predicted Outcome at 10th Percentile | Predicted Outcome at 90th Percentile | Coefficient (95% CI) | P-value | FDR Corrected P-value^b^ | Predicted Outcome at 10th Percentile | Predicted Outcome at 90th Percentile | Coefficient (95% CI) | P-value | FDR Corrected P-value^b^ |  |
| Telomere length at Year 1 (T/S ratio) | LAZ Year 1 | 660 | 1.2 | 1.7 | -1.46 | -1.15 | 0.31 (0.11, 0.5) | 0.00 | 0.00 | -1.54 | -1.31 | 0.23 (0.05, 0.42) | 0.01 | 0.01 |  |
|  | WAZ Year 1 | 662 | 1.2 | 1.7 | -1.22 | -1.13 | 0.09 (-0.12, 0.3) | 0.42 | 0.42 | -1.21 | -1.20 | 0.01 (-0.2, 0.23) | 0.91 | 0.91 |  |
|  | WLZ Year 1 | 660 | 1.2 | 1.7 | -0.71 | -0.81 | -0.1 (-0.3, 0.1) | 0.34 | 0.34 | -0.68 | -0.83 | -0.16 (-0.36, 0.05) | 0.14 | 0.14 |  |
|  | HCZ Year 1 | 660 | 1.2 | 1.7 | -1.76 | -1.75 | 0 (-0.18, 0.19) | 0.97 | 0.97 | -1.82 | -1.81 | 0.01 (-0.18, 0.2) | 0.94 | 0.94 |  |
|  | LAZ Year 2 | 580 | 1.2 | 1.7 | -1.45 | -1.24 | 0.21 (0.03, 0.39) | 0.02 | 0.03 | -1.51 | -1.45 | 0.06 (-0.04, 0.16) | 0.22 | 0.34 |  |
|  | WAZ Year 2 | 582 | 1.2 | 1.7 | -1.48 | -1.44 | 0.04 (-0.14, 0.21) | 0.69 | 0.69 | -1.77 | -1.70 | 0.07 (-0.03, 0.17) | 0.18 | 0.45 |  |
|  | WLZ Year 2 | 580 | 1.2 | 1.7 | -0.92 | -1.04 | -0.12 (-0.29, 0.04) | 0.15 | 0.18 | -0.99 | -1.01 | -0.03 (-0.14, 0.09) | 0.68 | 0.68 |  |
|  | HCZ Year 2 | 580 | 1.2 | 1.7 | -1.77 | -1.80 | -0.03 (-0.21, 0.14) | 0.72 | 0.73 | -1.85 | -1.81 | 0.04 (-0.18, 0.25) | 0.73 | 0.98 |  |
|  | Change in LAZ between Year 1 and Year 2 | 580 | 1.2 | 1.7 | -0.01 | -0.07 | -0.06 (-0.15, 0.03) | 0.21 | 0.41 | -0.23 | -0.28 | -0.05 (-0.14, 0.04) | 0.29 | 0.59 |  |
|  | Change in WAZ between Year 1 and Year 2 | 582 | 1.2 | 1.7 | -0.26 | -0.27 | -0.01 (-0.11, 0.09) | 0.79 | 0.79 | -0.37 | -0.38 | 0 (-0.11, 0.1) | 0.94 | 0.94 |  |
|  | Change in WLZ between Year 1 and Year 2 | 580 | 1.2 | 1.7 | -0.27 | -0.25 | 0.02 (-0.11, 0.15) | 0.79 | 0.79 | -0.24 | -0.20 | 0.04 (-0.09, 0.17) | 0.56 | 0.56 |  |
|  | Change in HCZ between Year 1 and Year 2 | 578 | 1.2 | 1.7 | -0.04 | -0.04 | 0 (0, 0) | 1.00 | 1.00 | -0.16 | -0.11 | 0.06 (-0.05, 0.17) | 0.31 | 0.39 |  |
|  | Length velocity between Year 1 and Year 2 | 580 | 1.2 | 1.7 | 0.80 | 0.81 | 0.01 (-0.01, 0.03) | 0.50 | 0.50 | 0.80 | 0.80 | 0 (-0.02, 0.03) | 0.79 | 0.79 |  |
|  | Weight velocity between Year 1 and Year 2 | 582 | 1.2 | 1.7 | 0.15 | 0.15 | 0 (-0.01, 0.01) | 0.60 | 0.60 | 0.15 | 0.15 | 0 (-0.01, 0.01) | 0.74 | 0.74 |  |
|  | Head circumference velocity between Year 1 and Year 2 | 580 | 1.2 | 1.7 | 0.14 | 0.15 | 0.01 (0, 0.02) | 0.13 | 0.27 | 0.15 | 0.16 | 0.01 (0, 0.02) | 0.13 | 0.26 |  |
|  | | | | | | | | | | | | | | | |

N, 10th Percentile, and 90th Percentile are from the unadjusted analyses

## T/S ratio = unit for relative telomere length; LAZ = Length-for-age Z score; WAZ = Weight-for-age Z score;

## WLZ = Weight-for-length Z score; HCZ = Head circumference-for-age Z score

## ^a^Adjusted for pre-specified covariates: Child age, child sex, birth order, prior child length and weight measurements (included in Year 2 outcomes only), time between anthropometry measurements (included in growth velocity and change in growth measurements between Year 1 and Year 2 outcomes only), season of measurement, caregiver-reported diarrhoea, mother’s age, mother’s height, mother’s education level, mother’s Center for Epidemiologic Studies Depression Scale Revised (CESD-R) score, mother’s Perceived Stress Scale score, mother’s lifetime exposure to physical, sexual, and emotional intimate partner violence, household food insecurity, number of children <18 years in the household, number of individuals living in the compound, distance in minutes to the primary water source, household floor materials, household wall materials, household electricity, and household assets (wardrobe, table, chair, clock, khat, chouki, radio, television, refrigerator, bicycle, motorcycle, sewing machine, mobile phone, cattle, goats, and chickens), and treatment arm (control or N+WSH) (Supplementary file 1a).

## ^b^Adjusted for multiple testing (by controlling the false discovery rate) within each hypothesis using the Benjamini-Hochberg procedure.

**Supplementary file 1c. Association Between Telomere Length at Year 2 and Growth**

| Exposure | Outcome | N | 10th Percentile | 90th Percentile | Outcome, 90th Percentile v. 10th Percentile | | | | | | | | | |
| --- | --- | --- | --- | --- | --- | --- | --- | --- | --- | --- | --- | --- | --- | --- |
|  |  |  |  |  | Unadjusted | | | | | Adjusted^a^ | | | | |
|  |  |  |  |  | Predicted Outcome at 10th Percentile | Predicted Outcome at 90th Percentile | Coefficient (95% CI) | P-value | FDR Corrected P-value^b^ | Predicted Outcome at 10th Percentile | Predicted Outcome at 90th Percentile | Coefficient (95% CI) | P-value | FDR Corrected P-value^b^ |
| Telomere length at Year 2  (T/S ratio) | LAZ Year 2 | 713 | 1.1 | 1.7 | -1.45 | -1.2 | 0.25 (0.06, 0.45) | 0.01 | 0.03 | -1.68 | -1.6 | 0.08 (-0.03, 0.19) | 0.17 | 0.34 |
|  | WAZ Year 2 | 713 | 1.1 | 1.7 | -1.47 | -1.4 | 0.05 (-0.14, 0.25) | 0.59 | 0.69 | -1.84 | -1.8 | 0.06 (-0.05, 0.16) | 0.30 | 0.45 |
|  | WLZ Year 2 | 713 | 1.1 | 1.7 | -0.92 | -1.0 | -0.12 (-0.29, 0.05) | 0.17 | 0.18 | -0.98 | -1.1 | -0.08 (-0.2, 0.04) | 0.18 | 0.35 |
|  | HCZ Year 2 | 712 | 1.1 | 1.7 | -1.82 | -1.8 | 0.03 (-0.14, 0.21) | 0.73 | 0.73 | -1.94 | -1.9 | 0 (-0.18, 0.17) | 0.98 | 0.98 |
| N, 10th Percentile, and 90th Percentile are from the unadjusted analyses | | | | | | | | | | | | | | |

## T/S ratio = unit for relative telomere length; LAZ = Length-for-age Z score; WAZ = Weight-for-age Z score;

## WLZ = Weight-for-length Z score; HCZ = Head circumference-for-age Z score

## ^a^Adjusted for pre-specified covariates: Child age, child sex, birth order, prior child length and weight measurements from Year 1, season of measurement, caregiver-reported diarrhoea, mother’s age, mother’s height, mother’s education level, mother’s Center for Epidemiologic Studies Depression Scale Revised (CESD-R) score, mother’s Perceived Stress Scale score, mother’s lifetime exposure to physical, sexual, and emotional intimate partner violence, household food insecurity, number of children <18 years in the household, number of individuals living in the compound, distance in minutes to the primary water source, household floor materials, household wall materials, household electricity, and household assets (wardrobe, table, chair, clock, khat, chouki, radio, television, refrigerator, bicycle, motorcycle, sewing machine, mobile phone, cattle, goats, and chickens), and treatment arm (control or N+WSH) (Supplementary file 1a).

## ^b^Adjusted for multiple testing (by controlling the false discovery rate) within each hypothesis using the Benjamini-Hochberg procedure.

**Supplementary file 1d. Association Between Change in Telomere Length and Growth**

| Exposure | Outcome | N | 10th Percentile | 90th Percentile | Outcome, 90th Percentile v. 10th Percentile | | | | | | | | | | |
| --- | --- | --- | --- | --- | --- | --- | --- | --- | --- | --- | --- | --- | --- | --- | --- |
|  |  |  |  |  | Unadjusted | | | | | Adjusted^a^ | | | | | |
|  |  |  |  |  | Predicted Outcome at 10th Percentile | Predicted Outcome at 90th Percentile | Coefficient (95% CI) | P-value | FDR Corrected P-value^b^ | Predicted Outcome at 10th Percentile | Predicted Outcome at 90th Percentile | Coefficient (95% CI) | P-value | FDR Corrected P-value^b^ |  |
| Change in telomere length between Year 1 and Year 2 (T/S ratio) | LAZ Year 2 | 557 | -0.43 | 0.44 | -1.30 | -1.33 | -0.03 (-0.22, 0.15) | 0.74 | 0.74 | -1.47 | -1.50 | -0.03 (-0.13, 0.07) | 0.62 | 0.62 |  |
|  | WAZ Year 2 | 557 | -0.43 | 0.44 | -1.40 | -1.51 | -0.11 (-0.3, 0.07) | 0.24 | 0.69 | -1.72 | -1.76 | -0.03 (-0.13, 0.07) | 0.53 | 0.53 |  |
|  | WLZ Year 2 | 557 | -0.43 | 0.44 | -0.93 | -1.05 | -0.12 (-0.29, 0.05) | 0.18 | 0.18 | -0.98 | -1.05 | -0.07 (-0.18, 0.04) | 0.24 | 0.35 |  |
|  | HCZ Year 2 | 557 | -0.43 | 0.44 | -1.76 | -1.82 | -0.07 (-0.25, 0.11) | 0.45 | 0.73 | -1.83 | -1.90 | -0.08 (-0.24, 0.09) | 0.36 | 0.98 |  |
|  | Change in LAZ between Year 1 and Year 2 | 557 | -0.43 | 0.44 | -0.04 | -0.05 | -0.01 (-0.1, 0.09) | 0.89 | 0.89 | -0.23 | -0.23 | 0 (-0.09, 0.08) | 1.00 | 1.00 |  |
|  | Change in WAZ between Year 1 and Year 2 | 557 | -0.43 | 0.44 | -0.26 | -0.28 | -0.01 (-0.11, 0.08) | 0.79 | 0.79 | -0.36 | -0.40 | -0.04 (-0.13, 0.05) | 0.36 | 0.73 |  |
|  | Change in WLZ between Year 1 and Year 2 | 557 | -0.43 | 0.44 | -0.26 | -0.28 | -0.02 (-0.15, 0.11) | 0.74 | 0.79 | -0.23 | -0.27 | -0.04 (-0.16, 0.08) | 0.53 | 0.56 |  |
|  | Change in HCZ between Year 1 and Year 2 | 555 | -0.43 | 0.44 | -0.01 | -0.07 | -0.06 (-0.16, 0.04) | 0.26 | 0.53 | -0.14 | -0.19 | -0.05 (-0.15, 0.06) | 0.39 | 0.39 |  |
|  | Length velocity between Year 1 and Year 2 | 557 | -0.43 | 0.44 | 0.81 | 0.80 | -0.01 (-0.03, 0.01) | 0.49 | 0.50 | 0.84 | 0.82 | -0.01 (-0.03, 0.01) | 0.29 | 0.58 |  |
|  | Weight velocity between Year 1 and Year 2 | 557 | -0.43 | 0.44 | 0.15 | 0.15 | 0 (-0.01, 0) | 0.39 | 0.60 | 0.15 | 0.15 | 0 (-0.01, 0.01) | 0.48 | 0.74 |  |
|  | Head circumference velocity between Year 1 and Year 2 | 557 | -0.43 | 0.44 | 0.14 | 0.14 | -0.01 (-0.02, 0.01) | 0.37 | 0.37 | 0.16 | 0.15 | -0.01 (-0.02, 0.01) | 0.35 | 0.35 |  |
|  | | | | | | | | | | | | | | | |

N, 10th Percentile, and 90th Percentile are from the unadjusted analyses

T/S ratio = unit for relative telomere length; LAZ = Length-for-age Z score; WAZ = Weight-for-age Z score;

WLZ = Weight-for-length Z score; HCZ = Head circumference-for-age Z score

^a^Adjusted for pre-specified covariates: Child age, child sex, birth order, prior child length and weight measurements from Year 1 (included in Year 2 outcomes only), season of measurement, time between anthropometry measurements (included in growth velocity and change in growth measurements between Year 1 and Year 2 outcomes only), caregiver-reported diarrhoea, mother’s age, mother’s height, mother’s education level, mother’s Center for Epidemiologic Studies Depression Scale Revised (CESD-R) score, mother’s Perceived Stress Scale score, mother’s lifetime exposure to physical, sexual, and emotional intimate partner violence, household food insecurity, number of children <18 years in the household, number of individuals living in the compound, distance in minutes to the primary water source, household floor materials, household wall materials, household electricity, and household assets (wardrobe, table, chair, clock, khat, chouki, radio, television, refrigerator, bicycle, motorcycle, sewing machine, mobile phone, cattle, goats, and chickens), and treatment arm (control or N+WSH) (Supplementary file 1a).

^b^Adjusted for multiple testing (by controlling the false discovery rate) within each hypothesis using the Benjamini-Hochberg procedure.

**Supplementary file 1e. Post-hoc Analyses: Association Between Growth at Month 3 and Subsequent Telomere Length**

| Exposure | Outcome | N | 10th Percentile | 90th Percentile | Outcome, 90th Percentile v. 10th Percentile | | | | | | | | | | |
| --- | --- | --- | --- | --- | --- | --- | --- | --- | --- | --- | --- | --- | --- | --- | --- |
|  |  |  |  |  | Unadjusted | | | | | Adjusted^a^ | | | | | |
|  |  |  |  |  | Predicted Outcome at 10th Percentile | Predicted Outcome at 90th Percentile | Coefficient (95% CI) | P-value | FDR Corrected P-value^b^ | Predicted Outcome at 10th Percentile | Predicted Outcome at 90th Percentile | Coefficient (95% CI) | P-value | FDR Corrected P-value^b^ |  |
| LAZ Month 3 | Telomere length at Year 1 (T/S ratio) | 413 | -2.82 | 0.02 | 1.45 | 1.44 | -0.01 (-0.1, 0.08) | 0.85 | 0.94 | 1.42 | 1.41 | -0.01 (-0.09, 0.08) | 0.9 | 0.95 |  |
|  | Telomere length at Year 2 (T/S ratio) | 423 | -2.82 | 0.08 | 1.43 | 1.47 | 0.03 (-0.02, 0.09) | 0.25 | 0.99 | 1.5 | 1.51 | 0.01 (-0.05, 0.07) | 0.7 | 0.94 |  |
|  |  |  |  |  |  |  |  |  |  |  |  |  |  |  |  |
| WAZ Month 3 | Telomere length at Year 1 (T/S ratio) | 414 | -2.66 | -0.02 | 1.44 | 1.44 | -0.01 (-0.06, 0.05) | 0.86 | 0.94 | 1.42 | 1.4 | -0.02 (-0.08, 0.03) | 0.42 | 0.84 |  |
|  | Telomere length at Year 2 (T/S ratio) | 423 | -2.74 | -0.02 | 1.43 | 1.45 | 0.01 (-0.05, 0.08) | 0.71 | 0.99 | 1.5 | 1.5 | 0 (-0.07, 0.06) | 0.94 | 0.94 |  |
|  |  |  |  |  |  |  |  |  |  |  |  |  |  |  |  |
| WLZ Month 3 | Telomere length at Year 1 (T/S ratio) | 411 | -1.59 | 1.18 | 1.45 | 1.42 | -0.03 (-0.09, 0.02) | 0.26 | 0.71 | 1.43 | 1.39 | -0.03 (-0.09, 0.02) | 0.2 | 0.74 |  |
|  | Telomere length at Year 2 (T/S ratio) | 421 | -1.59 | 1.17 | 1.47 | 1.43 | -0.05 (-0.1, 0.01) | 0.11 | 0.64 | 1.51 | 1.48 | -0.03 (-0.09, 0.02) | 0.27 | 0.94 |  |
|  |  |  |  |  |  |  |  |  |  |  |  |  |  |  |  |
| HCZ Month 3 | Telomere length at Year 1 (T/S ratio) | 412 | -3.1 | -0.38 | 1.44 | 1.44 | 0 (-0.06, 0.06) | 0.99 | 0.99 | 1.42 | 1.41 | -0.01 (-0.07, 0.05) | 0.77 | 0.95 |  |
|  | Telomere length at Year 2 (T/S ratio) | 419 | -3.1 | -0.42 | 1.43 | 1.45 | 0.02 (-0.04, 0.09) | 0.5 | 0.99 | 1.49 | 1.5 | 0.01 (-0.05, 0.07) | 0.74 | 0.94 |  |
|  | | | | | | | | | | | | | | | |

N, 10th Percentile, and 90th Percentile are from the unadjusted analyses.

T/S ratio = unit for relative telomere length; LAZ = Length-for-age Z score; WAZ = Weight-for-age Z score;

WLZ = Weight-for-length Z score; HCZ = Head circumference-for-age Z score

^a^Adjusted for pre-screened covariates: Child age, child sex, birth order, mother’s age, mother’s height, mother’s education, mother’s Perceived Stress Scale score, mother’s Center for Epidemiological Studies Depression Scale Revised (CESD-R) score, mother’s lifetime exposure to physical, sexual, and emotional intimate partner violence, household food insecurity, number of individuals <18 years old in household, number of individuals in compound, distance to primary water source, household floor materials, household wall materials, household assets (electricity, wardrobe, table, chair, clock, khat, chouki, radio, television, refrigerator, bicycle, motorcycle, sewing machine, mobile phone, cattle, goats, chickens), season of measurement, treatment arm (control or N+WSH), and caregiver-reported diarrhoea (Supplementary file 1a).

^b^Adjusted for multiple testing (by controlling the false discovery rate) within each hypothesis using the Benjamini-Hochberg procedure.

**Supplementary file 1f. Post-hoc Analyses: Association Between Growth at Year 1 and Subsequent Telomere Length**

| Exposure | Outcome | N | 10th Percentile | 90th Percentile | Outcome, 90th Percentile v. 10th Percentile | | | | | | | | | | |
| --- | --- | --- | --- | --- | --- | --- | --- | --- | --- | --- | --- | --- | --- | --- | --- |
|  |  |  |  |  | Unadjusted | | | | | Adjusted^a^ | | | | | |
|  |  |  |  |  | Predicted Outcome at 10th Percentile | Predicted Outcome at 90th Percentile | Coefficient (95% CI) | P-value | FDR Corrected P-value^b^ | Predicted Outcome at 10th Percentile | Predicted Outcome at 90th Percentile | Coefficient (95% CI) | P-value | FDR Corrected P-value^b^ |  |
| LAZ Year 1 | Telomere length at Year 2 (T/S ratio) | 617 | -2.76 | -0.14 | 1.39 | 1.45 | 0.06 (0.01, 0.11) | 0.01 | 0.3 | 1.41 | 1.46 | 0.04 (-0.01, 0.1) | 0.09 | 0.94 |  |
|  |  |  |  |  |  |  |  |  |  |  |  |  |  |  |  |
| WAZ Year 1 | Telomere length at Year 2 (T/S ratio) | 617 | -2.68 | -0.01 | 1.43 | 1.43 | 0 (-0.05, 0.05) | 0.91 | 0.99 | 1.44 | 1.42 | -0.02 (-0.07, 0.03) | 0.45 | 0.94 |  |
|  |  |  |  |  |  |  |  |  |  |  |  |  |  |  |  |
| WLZ Year 1 | Telomere length at Year 2 (T/S ratio) | 617 | -2.1 | 0.39 | 1.45 | 1.41 | -0.04 (-0.09, 0.01) | 0.1 | 0.64 | 1.48 | 1.42 | -0.06 (-0.11, -0.01) | 0.02 | 0.53 |  |
|  |  |  |  |  |  |  |  |  |  |  |  |  |  |  |  |
| HCZ Year 1 | Telomere length at Year 2 (T/S ratio) | 613 | -2.91 | -0.6 | 1.42 | 1.42 | 0 (-0.05, 0.05) | 0.96 | 0.99 | 1.52 | 1.5 | -0.02 (-0.07, 0.03) | 0.42 | 0.94 |  |
|  | | | | | | | | | | | | | | | |

N, 10th Percentile, and 90th Percentile are from the unadjusted analyses.

T/S ratio = unit for relative telomere length; LAZ = Length-for-age Z score; WAZ = Weight-for-age Z score;

WLZ = Weight-for-length Z score; HCZ = Head circumference-for-age Z score

^a^Adjusted for pre-screened covariates: Child age, child sex, birth order, mother’s age, mother’s height, mother’s education, mother’s Perceived Stress Scale score, mother’s Center for Epidemiological Studies Depression Scale Revised (CESD-R) score, mother’s lifetime exposure to physical, sexual, and emotional intimate partner violence, household food insecurity, number of individuals <18 years old in household, number of individuals in compound, distance to primary water source, household floor materials, household wall materials, household assets (electricity, wardrobe, table, chair, clock, khat, chouki, radio, television, refrigerator, bicycle, motorcycle, sewing machine, mobile phone, cattle, goats, chickens), season of measurement, treatment arm (control or N+WSH), and caregiver-reported diarrhoea (Supplementary file 1a).

^b^Adjusted for multiple testing (by controlling the false discovery rate) within each hypothesis using the Benjamini-Hochberg procedure.

**Supplementary file 1g. Post-hoc Analyses: Association Between Change in Growth and Telomere Length**

| Exposure | Outcome | N | 10th Percentile | 90th Percentile | Outcome, 90th Percentile v. 10th Percentile | | | | | | | | | | |
| --- | --- | --- | --- | --- | --- | --- | --- | --- | --- | --- | --- | --- | --- | --- | --- |
|  |  |  |  |  | Unadjusted | | | | | Adjusted^a^ | | | | | |
|  |  |  |  |  | Predicted Outcome at 10th Percentile | Predicted Outcome at 90th Percentile | Coefficient (95% CI) | P-value | FDR Corrected P-value | Predicted Outcome at 10th Percentile | Predicted Outcome at 90th Percentile | Coefficient (95% CI) | P-value | FDR Corrected P-value^b^ |  |
| Change in LAZ between Month 3 and Year 1 | Telomere length at Year 1 (T/S ratio) | 412 | -1.25 | 0.99 | 1.39 | 1.48 | 0.08 (0.01, 0.16) | 0.03 | 0.31 | 1.37 | 1.44 | 0.06 (0, 0.13) | 0.05 | 0.51 |  |
|  | Telomere length at Year 2 (T/S ratio) | 391 | -1.12 | 0.99 | 1.44 | 1.46 | 0.03 (-0.02, 0.08) | 0.32 | 0.99 | 1.42 | 1.44 | 0.02 (-0.02, 0.07) | 0.32 | 0.94 |  |
| Change in WAZ between Month 3 and Year 1 | Telomere length at Year 1 (T/S ratio) | 414 | -1.11 | 0.78 | 1.42 | 1.45 | 0.02 (-0.03, 0.08) | 0.43 | 0.89 | 1.4 | 1.43 | 0.03 (-0.03, 0.08) | 0.32 | 0.84 |  |
|  | Telomere length at Year 2 (T/S ratio) | 391 | -1.11 | 0.85 | 1.38 | 1.37 | -0.01 (-0.1, 0.08) | 0.83 | 0.99 | 1.35 | 1.36 | 0 (-0.08, 0.09) | 0.93 | 0.94 |  |
| Change in WLZ between Month 3 and Year 1 | Telomere length at Year 1 (T/S ratio) | 410 | -1.99 | 0.65 | 1.38 | 1.41 | 0.03 (-0.06, 0.12) | 0.49 | 0.89 | 1.36 | 1.39 | 0.02 (-0.06, 0.11) | 0.61 | 0.84 |  |
|  | Telomere length at Year 2 (T/S ratio) | 389 | -1.99 | 0.65 | 1.42 | 1.47 | 0.06 (-0.01, 0.12) | 0.1 | 0.64 | 1.42 | 1.43 | 0.02 (-0.04, 0.08) | 0.55 | 0.94 |  |
| Change in HCZ between Month 3 and Year 1 | Telomere length at Year 1 (T/S ratio) | 411 | -0.95 | 1.05 | 1.43 | 1.45 | 0.01 (-0.04, 0.07) | 0.67 | 0.92 | 1.4 | 1.42 | 0.02 (-0.04, 0.08) | 0.57 | 0.84 |  |
|  | Telomere length at Year 2 (T/S ratio) | 384 | -0.96 | 1.06 | 1.46 | 1.44 | -0.02 (-0.08, 0.04) | 0.58 | 0.99 | 1.44 | 1.41 | -0.03 (-0.1, 0.03) | 0.32 | 0.94 |  |
|  |  |  |  |  |  |  |  |  |  |  |  |  |  |  |  |
| Change in LAZ between Year 1 and Year 2 | Telomere length at Year 2 (T/S ratio) | 617 | -0.76 | 0.42 | 1.44 | 1.42 | -0.02 (-0.07, 0.02) | 0.35 | 0.99 | 1.45 | 1.43 | -0.02 (-0.07, 0.03) | 0.45 | 0.94 |  |
| Change in WAZ between Year 1 and Year 2 | Telomere length at Year 2 (T/S ratio) | 617 | -0.82 | 0.4 | 1.43 | 1.42 | 0 (-0.05, 0.04) | 0.88 | 0.99 | 1.44 | 1.44 | 0 (-0.04, 0.05) | 0.87 | 0.94 |  |
| Change in WLZ between Year 1 and Year 2 | Telomere length at Year 2 (T/S ratio) | 617 | -0.9 | 0.74 | 1.43 | 1.43 | 0 (-0.04, 0.05) | 0.97 | 0.99 | 1.44 | 1.44 | 0 (-0.04, 0.05) | 0.86 | 0.94 |  |
| Change in HCZ between Year 1 and Year 2 | Telomere length at Year 2 (T/S ratio) | 613 | -0.65 | 0.59 | 1.43 | 1.43 | 0 (-0.05, 0.05) | 0.99 | 0.99 | 1.51 | 1.52 | 0.01 (-0.04, 0.06) | 0.65 | 0.94 |  |
|  |  |  |  |  |  |  |  |  |  |  |  |  |  |  |  |
| Change in LAZ between Month 3 and Year 2 | Telomere length at Year 2 (T/S ratio) | 423 | -1.43 | 0.98 | 1.44 | 1.46 | 0.02 (-0.03, 0.07) | 0.46 | 0.99 | 1.49 | 1.52 | 0.03 (-0.03, 0.08) | 0.34 | 0.94 |  |
| Change in WAZ between Month 3 and Year 2 | Telomere length at Year 2 (T/S ratio) | 423 | -1.36 | 0.84 | 1.43 | 1.43 | 0 (-0.07, 0.07) | 0.97 | 0.99 | 1.5 | 1.51 | 0.01 (-0.05, 0.07) | 0.68 | 0.94 |  |
| Change in WLZ between Month 3 and Year 2 | Telomere length at Year 2 (T/S ratio) | 421 | -1.97 | 0.69 | 1.45 | 1.45 | 0.01 (-0.05, 0.07) | 0.76 | 0.99 | 1.5 | 1.5 | 0 (-0.06, 0.05) | 0.94 | 0.94 |  |
| Change in HCZ between Month 3 and Year 2 | Telomere length at Year 2 (T/S ratio) | 418 | -1.16 | 0.98 | 1.46 | 1.44 | -0.02 (-0.09, 0.04) | 0.52 | 0.99 | 1.51 | 1.49 | -0.02 (-0.09, 0.04) | 0.52 | 0.94 |  |
|  | | | | | | | | | | | | | | | |

N, 10th Percentile, and 90th Percentile are from the unadjusted analyses.

T/S ratio = unit for relative telomere length; LAZ = Length-for-age Z score; WAZ = Weight-for-age Z score;

WLZ = Weight-for-length Z score; HCZ = Head circumference-for-age Z score

^a^Adjusted for pre-screened covariates: Child age, child sex, birth order, mother’s age, mother’s height, mother’s education, mother’s Perceived Stress Scale score, mother’s Center for Epidemiological Studies Depression Scale Revised (CESD-R) score, mother’s lifetime exposure to physical, sexual, and emotional intimate partner violence, household food insecurity, number of individuals <18 years old in household, number of individuals in compound, distance to primary water source, household floor materials, household wall materials, household assets (electricity, wardrobe, table, chair, clock, khat, chouki, radio, television, refrigerator, bicycle, motorcycle, sewing machine, mobile phone, cattle, goats, chickens), season of measurement, treatment arm (control or N+WSH), and caregiver-reported diarrhoea (Supplementary file 1a).

^b^Adjusted for multiple testing (by controlling the false discovery rate) within each hypothesis using the Benjamini-Hochberg procedure.

**Supplementary file 1h. Post-hoc Analyses: Association Between Growth Velocity and Telomere Length**

| Exposure | Outcome | N | 10th Percentile | 90th Percentile | Outcome, 90th Percentile v. 10th Percentile | | | | | | | | | |
| --- | --- | --- | --- | --- | --- | --- | --- | --- | --- | --- | --- | --- | --- | --- |
|  |  |  |  |  | Unadjusted | | | | | Adjusted^a^ | | | | |
|  |  |  |  |  | Predicted Outcome at 10th Percentile | Predicted Outcome at 90th Percentile | Coefficient (95% CI) | P-value | FDR Corrected P-value | Predicted Outcome at 10th Percentile | Predicted Outcome at 90th Percentile | Coefficient (95% CI) | P-value | FDR Corrected P-value^b^ |
| Length velocity between Month 3 and Year 1 (cm/month) | Telomere length at Year 1 (T/S ratio) | 414 | 1.08 | 1.82 | 1.45 | 1.43 | -0.01 (-0.07, 0.04) | 0.65 | 0.92 | 1.39 | 1.44 | 0.06 (-0.02, 0.13) | 0.15 | 0.74 |
|  | Telomere length at Year 2 (T/S ratio) | 392 | 1.09 | 1.81 | 1.45 | 1.45 | 0 (-0.07, 0.06) | 0.9 | 0.99 | 1.38 | 1.43 | 0.06 (-0.04, 0.15) | 0.26 | 0.94 |
| Weight velocity between Month 3 and Year 1 (kg/month) | Telomere length at Year 1 (T/S ratio) | 415 | 0.17 | 0.42 | 1.46 | 1.42 | -0.04 (-0.1, 0.02) | 0.18 | 0.67 | 1.4 | 1.42 | 0.02 (-0.06, 0.11) | 0.6 | 0.84 |
|  | Telomere length at Year 2 (T/S ratio) | 392 | 0.17 | 0.42 | 1.46 | 1.44 | -0.02 (-0.09, 0.04) | 0.45 | 0.99 | 1.42 | 1.44 | 0.03 (-0.06, 0.12) | 0.58 | 0.94 |
| Head circumference velocity between Month 3 and Year 1 (cm/month) | Telomere length at Year 1 (T/S ratio) | 415 | 0.33 | 0.76 | 1.47 | 1.41 | -0.06 (-0.11, 0) | 0.06 | 0.32 | 1.41 | 1.41 | 0 (-0.08, 0.09) | 0.95 | 0.95 |
|  | Telomere length at Year 2 (T/S ratio) | 392 | 0.34 | 0.77 | 1.47 | 1.42 | -0.04 (-0.1, 0.02) | 0.15 | 0.72 | 1.46 | 1.39 | -0.07 (-0.18, 0.05) | 0.24 | 0.94 |
|  |  |  |  |  |  |  |  |  |  |  |  |  |  |  |
| Length velocity between Year 1 and Year 2 (cm/month) | Telomere length at Year 2 (T/S ratio) | 617 | 0.66 | 0.94 | 1.43 | 1.43 | 0.01 (-0.04, 0.06) | 0.75 | 0.99 | 1.45 | 1.44 | -0.01 (-0.07, 0.04) | 0.62 | 0.94 |
| Weight velocity between Year 1 and Year 2 (kg/month) | Telomere length at Year 2 (T/S ratio) | 617 | 0.1 | 0.2 | 1.43 | 1.42 | -0.01 (-0.07, 0.04) | 0.66 | 0.99 | 1.46 | 1.43 | -0.02 (-0.08, 0.03) | 0.42 | 0.94 |
| Head circumference velocity between Year 1 and Year 2 (cm/month) | Telomere length at Year 2 (T/S ratio) | 617 | 0.09 | 0.21 | 1.42 | 1.43 | 0.01 (-0.03, 0.05) | 0.59 | 0.99 | 1.44 | 1.45 | 0.01 (-0.03, 0.05) | 0.66 | 0.94 |
|  |  |  |  |  |  |  |  |  |  |  |  |  |  |  |
| Length velocity between Month 3 and Year 2 (cm/month) | Telomere length at Year 2 (T/S ratio) | 424 | 0.89 | 1.29 | 1.45 | 1.45 | 0 (-0.06, 0.06) | 0.89 | 0.99 | 1.47 | 1.53 | 0.06 (-0.02, 0.14) | 0.12 | 0.94 |
| Weight velocity between Month 3 and Year 2 (kg/month) | Telomere length at Year 2 (T/S ratio) | 424 | 0.16 | 0.28 | 1.47 | 1.44 | -0.03 (-0.09, 0.03) | 0.34 | 0.99 | 1.5 | 1.51 | 0.01 (-0.06, 0.08) | 0.77 | 0.94 |
| Head circumference velocity between Month 3 and Year 2 (cm/month) | Telomere length at Year 2 (T/S ratio) | 424 | 0.23 | 0.43 | 1.47 | 1.42 | -0.05 (-0.11, 0.01) | 0.11 | 0.64 | 1.51 | 1.5 | -0.01 (-0.11, 0.09) | 0.85 | 0.94 |
|  | | | | | | | | | | | | | | |

N, 10th Percentile, and 90th Percentile are from the unadjusted analyses.

T/S ratio = unit for relative telomere length

^a^Adjusted for pre-screened covariates: Child age, child sex, birth order, mother’s age, mother’s height, mother’s education, mother’s Perceived Stress Scale score, mother’s Center for Epidemiological Studies Depression Scale Revised (CESD-R) score, mother’s lifetime exposure to physical, sexual, and emotional intimate partner violence, household food insecurity, number of individuals <18 years old in household, number of individuals in compound, distance to primary water source, household floor materials, household wall materials, household assets (electricity, wardrobe, table, chair, clock, khat, chouki, radio, television, refrigerator, bicycle, motorcycle, sewing machine, mobile phone, cattle, goats, chickens), season of measurement, treatment arm (control or N+WSH), and caregiver-reported diarrhoea (Supplementary file 1a).

^b^Adjusted for multiple testing (by controlling the false discovery rate) within each hypothesis using the Benjamini-Hochberg procedure.

**References**

1. Luby SP, Rahman M, Arnold BF, et al. Effects of water quality, sanitation, handwashing, and nutritional interventions on diarrhoea and child growth in rural Bangladesh: a cluster randomised controlled trial. *Lancet Glob Health* 2018; **6**: e302-e15.

2. Black MM, Baqui AH, Zaman K, et al. Depressive symptoms among rural Bangladeshi mothers: implications for infant development. *J Child Psychol Psychiatry* 2007; **48**: 764-72.

3. Cohen S, Kamarck T, Mermelstein R. A global measure of perceived stress. *J Health Soc Behav* 1983; **24**: 385-96.

4. Cohen S. *Perceived Stress Scale*. 1994 [cited 2019 August 19]; Available from: http://www.mindgarden.com/documents/PerceivedStressScale.pdf

5. Garcia-Moreno C, Jansen, H.A., Ellsberg, M., Heise, L., Watts, C. *WHO multi-country study on women’s health and domestic violence against women. Initial results on prevalence, health outcomes, and women’s responses*. Geneva: World Health Organization; 2005.

6. Coates J, Swindale A, Bilinsky P. *Household Food Insecurity Access Scale (HFIAS) for Measurement of Food Access: Indicator Guide.* Washington, DC: Academy for Educational Development, Food and Nutrition Technical Assistance Project (FANTA); 2007.
